# Retraction Note: Curcumin inhibits proliferation, migration, invasion and promotes apoptosis of retinoblastoma cell lines through modulation of miR-99a and JAK/STAT pathway

**DOI:** 10.1186/s12885-024-12248-z

**Published:** 2024-04-15

**Authors:** Yaping Li, Weixuan Sun, Ning Han, Ying Zou, Dexin Yin

**Affiliations:** 1 Department of Ophthalmology, The Second Hospital of Jilin University, 130041 Changchun, Jilin China; 2 Department of Gastroenterological Surgery, China-Japan Union Hospital of Jilin University, 130033 Changchun, Jilin China; 3 Department of Vascular Surgery, China-Japan Union Hospital of Jilin University, No.126, Xiantai Street, 130033 Changchun, Jilin China

**Retraction Note:** ***BMC Cancer 18, 1230 (2018)***

10.1186/s12885-018-5130-y

The Editors have retracted this article following an investigation report by the Ministry of Science and Technology of the People’s Republic of China. Their investigation found attempts to tamper with the data. The Editors therefore no longer have confidence in the integrity of the data in this article.

None of the authors have responded to any correspondence from the editor/publisher about this retraction.

